# The Catalytic Function of Nonheme Iron (III) Complex for Hydrocarbon Oxidation

**DOI:** 10.1155/2010/861892

**Published:** 2010-06-24

**Authors:** Giorgos Bilis, Maria Louloudi

**Affiliations:** Department of Chemistry, University of Ioannina, 45110 Ioannina, Greece

## Abstract

A detailed catalytic study of LFe^III^Cl (where L = 3-{2-[2-(3-hydroxy-1,3-diphenyl-allylideneamino)-ethylamino]-ethylimino}-1,3-diphenyl-propen-1-ol) for hydrocarbon oxidation was carried out, focusing on the role of solvent, atmospheric dioxygen, and oxidant on catalytic efficiency. The data showed that LFe^III^Cl catalyst was efficient in homogeneous hydrocarbon oxidations providing significant yields. Moreover, *tert*-BuOOH provided comparable oxidation yields with H_2_O_2_, slightly favoring the formation of alcohols and ketones *versus* epoxides. Dioxygen intervened in the catalytic reaction, influencing the nature of oxidation products. The polarity of solvent strongly influenced the reaction rates and the nature of oxidation products. A mechanistic model is postulated assuming that LFe^III^Cl functions *via* the formation of iron-hydroperoxo-species, followed by a radical-based mechanistic path.

## 1. Introduction

Hydrocarbon oxidation, under mild and environmental friendly conditions, is an important research field, since industrial processes, especially in pharmaceutical industry, are based on the efficiency of direct and selective transformation of hydrocarbons in oxygen-containing products such as aldehydes, ketones alcohols, diols, and epoxides [1, 2]. However, selective oxidation of alkanes, under mild conditions, is a difficult task due to their chemical inertness. Nevertheless, in nature many iron enzymes activate dioxygen and catalyze the stereospesific oxidation of C=C or C–H bonds [3, 4]. Heme iron-proteins such as hemoglobin, myoglobin and cytochromes oxygenase [5] and nonheme enzymes such as methane monooxygenase [6, 7] and Rieske dioxygenases [8, 9] are able to oxidize hydrocarbons [10] *via* biochemical oxygen transport and electron-transfer reactions.

The objective to construct convenient artificial systems for efficient hydrocarbon oxidation using biomimetic iron complexes [11, 12] as catalysts [13–15] by activating green oxidants is particularly interesting. Bioinspired catalytic systems commonly use mild oxidants such as dioxygen, hydrogen peroxide, or tert-butyl hydrogen peroxide (TBHP) [16–20]. In this context, stereoselective hydroxylation, epoxidation, and *cis-*dihydroxylation by synthetic iron-biomimetic catalysts have been reported [21–29]. Iron-peroxo species are invoked to be part of the mechanism of several bioinspired oxidation catalysts [30, 31]. More particularly, iron-complexes react with H_2_O_2_ or alkyl hydroperoxides forming low-spin Fe^III^OOH [32, 33] or Fe^III^OOR [34, 35] which are the key-species in oxidation reactions [16]. 

Recently, we have reported the synthesis and characterization of the ligand 3-{2-[2-(3-hydroxy-1,3-diphenyl-allylideneamino)-ethylamino]-ethylimino}-1,3-diphenyl-propen-1-ol and its immobilization on silica surface *via* formation of covalent bridging between the ligand secondary amine and the silica OH-groups [36, 37]. Herein for brevity this ligand will be named **L**. The corresponding Mn(II) [36, 37] and Fe(III) [38] catalysts were shown to have remarkable catalytic activity in hydrocarbons oxidation using H_2_O_2_ as oxidant. 

In the present contribution, we report a detailed catalytic study of LFe^III^Cl focusing mainly on the influence of (a) atmospheric dioxygen, (b) solvent system, and (c) oxidant on its catalytic efficiency. The obtained information is compared with previous data of the LFe^III^Cl catalyst, and mechanistic aspects are also discussed.

## 2. Experimental

### 2.1. General

All substrates were purchased from Aldrich, in their highest commercial purity, stored at 5°C and purified by passage through a column of basic alumina prior to use. Hydrogen peroxide was 30% aqueous solution and tert-BuOOH was 5 M solution in decane. 

Infrared spectra were recorded on a Spectrum GX Perkin-Elmer FT-IR System. UV-Vis spectra were recorded using a UV/VIS/NIR JASCO Spectrophotometer. The iron amount was determined by Flame Atomic Absorption spectroscopy on a Perkin-Elmer AAS-700 spectrometer. Mössbauer spectra were recorded with a constant acceleration spectrometer using a ^57^Co (Rh) source at room temperature and a variable-temperature. X-band Electron Paramagnetic Resonance (EPR) spectra were recorded using a Brucker ER200D spectrometer at liquid N_2_ temperatures, equipped with an Agilent 5310A frequency counter. The spectrometer was running under a home-made software based on LabView described earlier [39]. Mass spectra were measured on a Agilent 1100 Series LC-MSD-Trap-SL spectrometer and solution. Thermogravimetric analyses were carried out using Shimadzu DTG-60 analyser. GC analysis was performed using an 8000 Fisons chromatograph with a flame ionization detector and a Shimadzu GC-17A gas chromatograph coupled with a GCMS-QP5000 mass spectrometer.

### 2.2. Catalysts Preparation and Characterization

#### 2.2.1. Preparation of LFe^III^Cl

To a stirred solution of ethanol and acetonitrile (15 ml) containing the ligand **L** = 3-{2-[2-(3-hydroxy-1,3-diphenyl-allylideneamino)-ethylamino]-ethylimino}-1,3-diphenyl-propen-1-ol [36, 37] a solution of FeCl_3_ in a mixture of EtOH and CH_3_CN was added slowly. The resulting mixture was stirred for 24 h at room temperature. Partially solvent evaporation resulted in separation of an orange solid product. The obtained LFe^III^Cl complex was washed with EtOH and CH_3_CN and dried under reduced pressure. 

#### 2.2.2. Characterization of LFe^III^Cl

Iron analysis by Flame Atomic Absorbance Spectroscopy and ESI-MS analysis indicated a molecular peak at *m/z* 605.2 that is attributed in [LFeCl + H]^+^ formation. The IR bands at 3297, 1591, and 1523 cm^−1^ were attributed to the *v*(NH), *v*(C=N), and *δ*(NH) vibrations. In the spectrum of the metal-free ligand **L**, the corresponding vibrations were detected at 3350, 1600, and 1535 cm^−1^; this shift indicates metal coordination to imine- and amine-nitrogen. In the IR spectra of **L** and the LFe^III^Cl the *v*(C–O) vibration was appeared at 1398 and 1389 cm^−1^, respectively, suggesting strong coordination of Fe to the enolic oxygen atoms of the ligand **L**. Information about the iron center was obtained by Electron Paramagnetic Resonance (EPR) and Mössbauer spectroscopy. At 78 K, the Mössbauer parameters are *δ* = 0.50 mm/s and Δ_EQ_ = 0.54 mm/s indicating an octahedral high-spin Fe^III^(S = 5/2) center, with the iron bound to nitrogen and oxygen atom donors [40]. The EPR spectrum of solid LFe^III^Cl is characerised by two peaks at g = 4.3 and 9.2, characteristic of a high-spin Fe^III^(S = 5/2) center in a rhombic ligand-field characterized by E/D ~ 0.33 [41]. The UV-Vis spectrum of LFe^III^Cl in CH_3_CN contains absorption bands at 282 (*ε* = 20800 M^−1^ cm^−1^) and 314 nm (*ε* = 25580 M^−1^ cm^−1^) due to intraligand transitions in the imine- and phenyl-groups [42]. The absorptions at 413 nm (*ε* = 7100 M^−1^ cm^−1^) and 512 nm (*ε* = 2000 M^−1^ cm^−1^) are attributed to LMCT [43]. The low-intensity 512 nm absorption can be attributed to charge transfer from ligand oxygen atoms to metal centre (p_*π*_ → Fe^III^ d_*π*_). Usually, a (p_*π*_ → Fe^III^ d_σ_*) transition is also observed at higher energy than the p_*π*_ → Fe^III^ d_*π*_ transition and could be related to the transition at 413 nm [35, 36]. The presence of Fe^III^-Cl bond also allows the presence of LMCT involving chloride p_*π*_ → Fe^III^ d_*π*_ orbitals [43, 44].

### 2.3. Catalytic Evaluation

#### 2.3.1. GC-MS

H_2_O_2_ or tert-BuOOH diluted in solvent (CH_3_CN or tert-amylalcohol) was slowly added (within a period of 5 min) to a solvent solution containing the catalyst and the substrate, at room temperature (25°C). For brevity in the text [H_2_O_2_ in tert-amylalcohol] will be referred as system A, [H_2_O_2_ in CH_3_CN] as system B, and [tert-BuOOH in CH_3_CN] as system C, respectively. As an internal standard, acetophenone or bromobenzene were used. Catalytic reactions were initiated by adding the oxidant into reaction mixture. 

The progress of the reaction was monitored by GC-MS, by removing small samples of the reaction mixture. The yields reported herein are based on the amount of oxidant H_2_O_2_ converted to oxygenated products. To establish the identity of the products unequivocally, the retention times and spectral data were compared to those of commercially available compounds. Blank experiments showed that, without catalyst, no oxidative reactions take place.

#### 2.3.2. Reaction Conditions

**(1) Optimization of [Oxidant : Substrate] Ratio**

**Figure 1:**
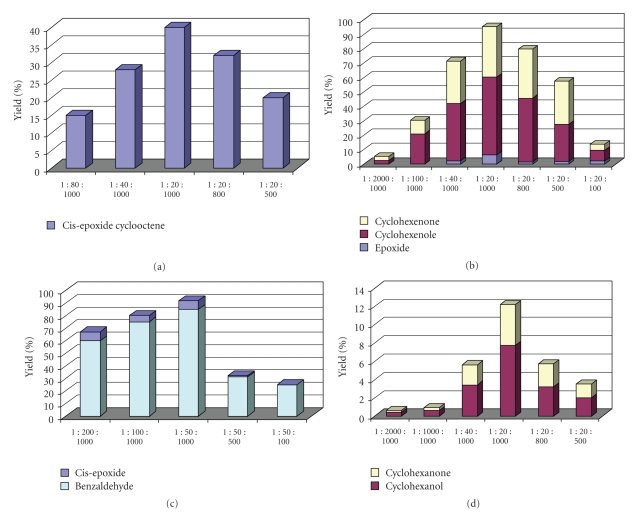
(a) Cis-cyclooctene oxidation is catalysed by LFe^III^Cl at varied Substrate-Oxidant molar ratio in CH_3_CN with H_2_O_2_. (b) Cyclohexene oxidation is catalysed by LFe^III^Cl at varied Substrate-Oxidant molar ratio in CH_3_CN with H_2_O_2_. (c) Styrene oxidation is catalysed by LFe^III^Cl at varied Substrate-Oxidant molar ratio in CH_3_CN with H_2_O_2_. (d) Cyclohexane oxidation is catalysed by LFe^III^Cl at varied Substrate-Oxidant molar ratio in CH_3_CN with H_2_O_2_. For all figures The given ratio is catalyst : oxidant : substrate.

To explore the optimum [oxidant-substrate] molar ratio, a first set of catalytic experiments was performed where the amount of substrate was kept constant and the amount of H_2_O_2_ was varied. Subsequently, a second set of catalytic experiments was run for varied amounts of substrate, using the optimum amount of H_2_O_2_ found in the first screening. For this screening, cyclooctene (Figure 1(a)), cyclohexene (Figure 1(b)), styrene (Figure 1(c)), and cyclohexane (Figure 1(d)) were tested. It is noted that H_2_O_2_ was diluted in acetonitrile (1/10 v/v) *prior* use, and it was introduced into the reaction mixture slowly.

Based on the data of Figures 1(a)–1(d), the higher oxidation yield was obtained by small amounts of H_2_O_2_ and large excess of substrate. This is consistent with the current view that (a) high oxidant concentration causes oxidative destruction of catalyst, (b) large excess of substrate protects the catalyst from oxidative degradation, and, moreover, (c) this substrate large excess minimizes the overoxidation of initial oxidation products [45].

Based on analogous experiments for all the substrates used in the present study, we found that for cyclohexene, methyl-cyclohexene, cyclooctene, limonene, and cyclohexane oxidation, the optimum molar ratio of [catalyst : oxidant : substrate] was equal to [1 : 20 : 1000] and for styrene, trans-*β*-methyl styrene and cis-stilbene oxidation, this molar ratio was equal to [1 : 50 : 1000].

**(2) Excess of Dioxygen**

**Figure 2:**
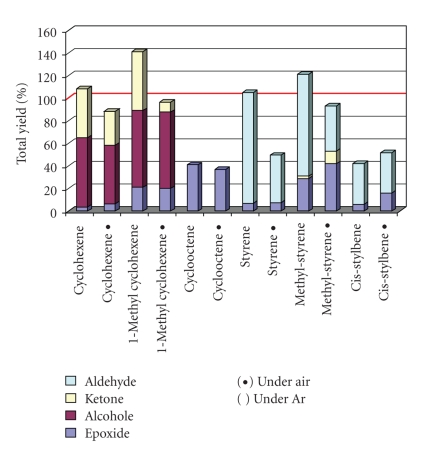
Bar chart representation of oxidations catalyzed by LFe^III^Cl with H_2_O_2_ in CH_3_CN under atmospheric air and under Ar.

Iron-based catalysts, in the presence of dioxygen, often adopt radical mechanistic paths. Thus, the LFe^III^Cl catalyst was evaluated in oxidation reactions with H_2_O_2_, (a) under atmospheric air *versus* (b) under inert Ar atmosphere (Table 1, Figure 2).

Based on the data of Table 1, it is observed that (a) the total yield of some oxidation reactions under air was over 100%; moreover, (b) the total oxidation yield under Ar was decreased. Especially, alcohol and ketone yields were strongly decreased under Ar. This suggests O_2_-involvement in the oxidation process which possibly propagates a radical autooxidation [46, 47] of the more reactive oxygen-containing products such as alcohols and ketones. Thus, to record reliable data, all catalytic experiments herein were performed under a vigorous Ar purge to avoid any trace of O_2_.

**(3) Solvent Effect on the Reaction Time**

**Figure 3:**
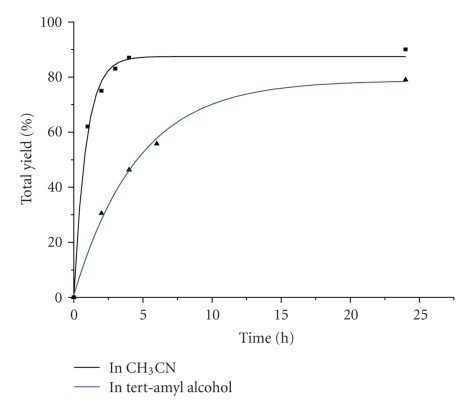
Time-dependent reaction profiles for cyclohexene oxidation catalysed by LFe^III^Cl with H_2_O_2_ in CH_3_CN and tert-amylalcohol.

The time course profiles of the LFe^III^Cl-catalysed oxidation of cyclohexene with H_2_O_2_ in CH_3_CN and in tert-amylalcohol are given in Figure 3. According to these data the catalytic reaction was practically accomplished within 4 h in CH_3_CN and within 12 h in *tert*-amylalcohol. Thus, the reaction rate is strongly influenced by solvent. This effect could be related to the solvent polarity. However, in both solvents, the total yield of cyclohexene oxidation is quite high providing 88.5% in CH_3_CN and 79.0% in *tert*-amylalcohol. It is noted that cyclohexene oxidation, in CH_3_CN with *tert*-BuOOH as oxidant, was complete within 2 h, resulting in a 76.3% total yield.

## 3. Results and Discussion

### 3.1. Hydrocarbon Oxidation by the Fe^III^-Catalysts

**Figure 4:**
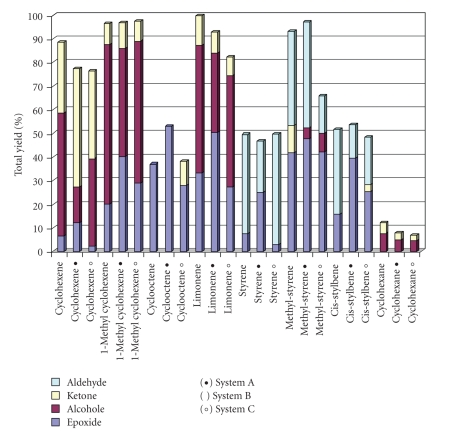
Distribution of oxidation products catalyzed by LFe^III^Cl, (a) in tert-amylalcohol with H_2_O_2_, (b) in CH_3_CN with H_2_O_2_, and (c) in CH_3_CN with tert-BuOOH. See Table 2 for further details.

The catalytic activity of LFe^III^Cl for hydrocarbon oxidation was evaluated using *tert*-BuOOH and H_2_O_2_ as oxidants in either CH_3_CN or *tert*-amylalcohol. Cyclohexene, methyl-cyclohexene, cyclooctene, limonene, and cyclohexane were used as substrates with a ratio of catalyst : oxidant : substrate equal to 1 : 20 : 1000 and styrene, trans-*β*-methyl styrene and cis-stilbene with a ratio of catalyst : oxidant : substrate equal to 1 : 50 : 1000. All oxidation reactions were carried out at room temperature under Ar atmosphere as described in details in Experimental Section. The obtained catalytic results are summarized in Table 2. Figure 4 provides a histogram-plot of the data of Table 2. The catalytic data of LFe^III^Cl with H_2_O_2_ in CH_3_CN were taken from [38] and are included here for comparison. 

Based on Table 2 we observe that cyclohexene and limonene oxidation catalyzed by the LFeCl provided oxidation products with a combined yield of 79.0% and 93.7%, respectively, in *tert*-amylalcohol with H_2_O_2_ (system A), 88.5% and 99.8% in CH_3_CN with H_2_O_2_ (system B), and 76.3% and 82.34% in CH_3_CN with tert-BuOOH (system C). Cyclohexene undergoes mainly allylic oxidation forming 2-cyclohexene-1-ol and 2-cyclohexene-1-one (with yields 16.0% and 50.7%, resp., by system A, 52.0% and 30.0% by system B, and 37.0% and 37.0% by system C). However cyclohexene epoxidation is also observed, providing low epoxide yields 12.3%, 6.5%, and 2.3% by systems A, B, and C, respectively. It is note that in *tert*-amylalcohol, the yield of epoxide and ketone increases, while the alcohol yield is reduced. 

The major products detected from limonene oxidation were (i) two epoxides (*cis*- and *trans*-) derived from epoxidation of the electron-rich double bond at 1,2- position and (ii) alcohols derived from hydroxylation of the double bond at 1- and 2-position and from hydroxylation at 6-position closed to 1,2-double bond. Oxidation products from the more accessible, though less electron-rich, double bond at 8,9-position were not observed. Additionally, considerable amounts of the corresponding ketone at 6-position were also formed. In summary, the yield (a) of limonene-epoxides (*cis*-1,2 and *trans*-1,2) was found to be 51.2% in system A, 33.3% in system B, and 27.4% in system C, (b) of limonene-alcohols (1-ol, 2-ol, and 6-ol) 33.5%, 54.0%, and 46.9%, respectively and (c) of 6-ketone 9.0%, 12.5%, and 8% in systems A, B, and C respectively. These data provide a total catalytic oxidation of limonene 93.7%, 99.8%, and 82.3% achieved by LFeCl, respectively, in *tert*-amylalcohol with H_2_O_2_ (system A), in CH_3_CN with H_2_O_2_ (system B), and in CH_3_CN with tert-BuOOH (system C). 

When methyl-cyclohexene was used as substrate, the detected oxidation products were *cis*-epoxide, 1-methyl-2-cyclohexen-1-ol, 3-methyl-2-cyclohexen-1-ol, and 3-methyl-2-cyclohexen-1-one. The corresponding yields found to be [40.0%, 20.0%, and 29.0%], [21.3%, 25.6%, and 23.7%], [24.5% 42.0%, and 36.3%], and [10.9%, 8.8%, and 8.5%] in catalytic systems A, B, and C, respectively. Generally, methyl-substituted alkenes are more reactive towards both epoxidation and allylic oxidation. Our findings confirm this aspect providing total yield of methyl-cyclohexene oxidation 93.7%, 98.8%, and 83.3% by the present catalytic systems.

*Cis-*cyclooctene as substrate afforded a single-product reaction with H_2_O_2_ catalysed by LFe^III^Cl resulting only in *cis*-cyclooctene epoxide with 53.0% and 37.0% yields in systems A and B, respectively. However, the use of tert-BuOOH as oxidant provided 28% epoxide and 10% 2-cyclooctenone. Generally, cyclooctene occurs more readily epoxydation than allylic oxidation; however, here with tert-BuOOH, overoxidized 2-cyclooctenone derived from allylic oxidation was also detected. 

*Cis*-stilbene oxidation in catalytic systems A and C provided *cis*- and *trans*- epoxides as major products [9.5% and 30% in system A] and [2.4% and 23.0% in C]. Benzaldehyde as oxidative cleavage product was also formed with yields 14% and 20%, respectively. In Catalytic system B, the detected products were benzaldehyde with yield 36.0% and *cis*-stilbene epoxide with yields 15.8%.

Styrene oxidation provided benzaldehyde as major product derived by oxidative cleavage of the exo-cyclic double bond with yields 20.0%, 35.0%, and 45.0% in systems A, B, and C, respectively. However, epoxide and phenyl acetaldehyde have been also formed by direct oxidation of the same double bond with [25.0% and 1.6%] in system A, [7.5% and 7.0%] in system B, and [3.0% and 1.6%] in system C. Overall, styrene was oxidised by LFe^III^Cl in different oxidation conditions providing total yields from 46.6% to 49.6%.

 The methyl-substituted styrene, *trans*-*β*-methyl styrene, is more reactive than styrene showing total oxidation yields 97.2%, 93.2%, and 65.8% in catalytic systems A, B, and C, respectively. The identified products were *trans*-epoxide (47.7%, 41.9%, and 42.0%) and benzaldehyde as oxidation cleavage adduct (45.0%, 40.0%, and 15.8%). In some cases, methyl-benzyl-ketone and methyl-benzyl-alcohol were also detected. 

Finally, cyclohexane oxidation by the present Fe^III^-catalyst, in the three A, B, and C catalytic conditions, gave cyclohexanol and cyclohexanone with combined yields 8.0%, 12.2%, and 7.0% while the corresponding alcohol/ketone (A/K) ratio was found to be 1.66, 1.75, and 1.71, respectively.

### 3.2. Mechanistic Considerations

The A/K ratio in cyclohexane can be used as a criterion of the presence and lifetime of free alkyl radical intermediates [31] as follows: (i) when A/K = 1, then we assume that the alkyl radicals are long-lived with a strong tendency to interact with O_2_ to form alkyl-peroxy-radicals [45]. At the end, following a Russell-type terminal stage [48], recombination of these radicals results in the formation of equimolar amounts of alcohol and ketone [21, 31]. (ii) When A/K > 1, the radical ^●^OH is formed by a metal-based system and the metal center reacts directly to form the corresponding alcohol. This is consistent with the formation of Fe^III^–OOH intermediate and the homolytic cleavage of O–O bond to ^●^HO radical and oxoiron(IV) followed by electrophilic addition of metal-based species to the substrate.

**Scheme 2:**
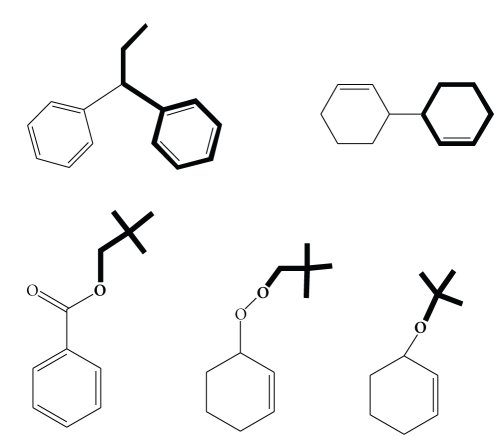
Schematic representation of detected byproducts.

Hydrocarbon oxidations catalyzed by LFe^III^Cl presented comparable selectivity and distribution of oxidation products, in the three experimental conditions studied herein. This implies similar mechanistic path in the catalysis. Generally, LFe^III^Cl is able to generate iron-hydroperoxo-species under the mild oxidation conditions used. It is known from other nonheme iron systems that Fe^III^–OOH could be either (a) a precursor or (b) itself an oxidant [22, 28, 46]. In the first case, possible homolytic cleavage of O–O bond leads to an Fe^IV^=O species and reactive ^●^HO radical while a heterolytic cleavage generates an Fe^v^=O and an OH^−^ species [22, 28, 46]. Herein, the major oxidation products of alkenes are alcohols and ketones, mainly derived by an allylic oxidation reaction. However, considerable amounts of epoxides have been also formed. Taking into account that the detected allylic oxidation products are the main component of the observed oxidation yield, a dominant radical mechanistic path is suggested. This suggestion is further supported by detection of traces of the compounds illustrated in Scheme 2. Their formation could be a result of (a) interaction between two allyl-radicals formed on substrates and (b) interaction of an allyl-radical on substrate and a hydro-peroxo radical. 

In this context, when tert-amylalcohol—a less polar solvent—was used with H_2_O_2_, the reaction time is considerably longer, the yield of the corresponding epoxides increased, and the yield of radical mechanism products, that is, alcohols and ketones decreases. On the other hand, the use of *tert*-BuOOH as oxidant enhanced the yield of the corresponding alcohols and ketones.

## 4. Conclusion

The LFe^III^Cl catalyst was efficient in homogeneous hydrocarbon oxidations providing significant yields. The catalytic experiments (a) were performed under inert argon atmosphere excluding any trace of O_2_ which favors radical oxidation paths and (b) included large excess of substrate, protecting the catalyst from oxidative degradation and minimizing the overoxidation of initial oxidation products. *tert*-BuOOH provided comparable oxidation yields with H_2_O_2_; nevertheless it slightly favors the formation of alcohols and ketones *versus* epoxides. The polarity of solvent strongly influences the reaction rate and the nature of oxidation products. We suggest that LFe^III^Cl functions *via* the formation of iron-hydroperoxo-species and the dominant mechanistic path is a radical one.

## Figures and Tables

**Scheme 1:**
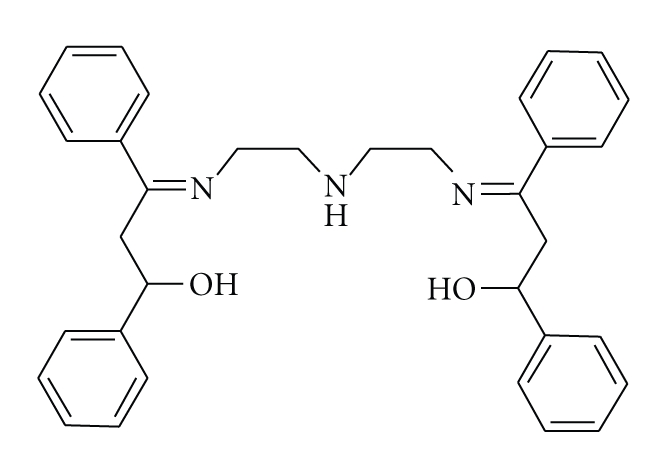
Schematic representation of ligand [3-{2-[2-(3-hydroxy-1,3-diphenyl-allylideneamino)-ethylamino]-ethylimino}-1,3-diphenyl-propen-1-ol] **(L).**

**Table 1:** Hydrocarbon oxidations catalyzed by LFeCl with H_2_O_2_ in CH_3_CN under atmospheric air and under Ar.

substrate	products	LFeCl under Ar^c^ Yield (%)	LFeCl under Ar^c^ Total yield (%)	LFeCl under air^c^ Yield (%)	LFeCl under air^c^ Total yield (%)
Cyclohexene^a^	cis-epoxide	6.5		3.5	
	2-cyclohexenol	52.0		61.5	
	2-cyclohexenone	30.0	88.5	43.2	108.2

1-Methyl-cyclohexene^a^	cis-epoxide	20.0		21.0	
	1-methyl-2-cyclohexen-1-ol	25.6		22.6	
	3-methyl-2-cyclohexen-1-ol	42.0		45.3	
	3-methyl-2-cyclohexen-1-one	8.8	96.4	52.0	140.9

Cyclooctene^a^	cis- epoxide	37.0	37.0	41.0	41.0

Styrene^b^	epoxide	7.5		7.0	
	phenyl-acetaldehyde	7.0		3.5	
	benzaldehyde	35.0	49.5	94.5	105.0

Methyl-Styrene^b^	trans-epoxide	41.9		29.0	
	methyl-benzyl-ketone	11.3		2.0	
	benzaldehyde	40.0	93.2	90.0	121.0

Cis- stylbene^b^	cis-epoxide	15.8		6.0	
	benzaldehyde	36.0	51.8	36.0	42.0

^a^Conditions: ratio of catalyst: H_2_O_2_: substrate= 1 : 20 : 1000. ^b^Conditions: ratio of catalyst : H_2_O_2_ : substrate = 1 : 50 : 1000. ^c^ Reactions were completed within 4 h.

**Table 2:** Hydrocarbon oxidation by LFe^III^Cl catalyst.

substrate	products	LFeCl^c^		LFeCl^d^		LFeCl^e^	
		Yield (%)	Total yield (%)	Yield (%)	Total yield (%)	Yield (%)	Total yield (%)
Cyclohexene^a^	cis-epoxide	12.3		6.5		2.3	
	2-cyclohexenol	16.0		52.0		37.0	
	2-cyclohexenone	50.7	79.0	30.0	88.5	37.0	76.3

	cis-epoxide	40.0		20.0		29.0	
1-Methyl-cyclohexene^a^	1-methyl-2-cyclohexen-1-ol	21.3		25.6		23.7	
	3-methyl-2-cyclohexen-1-ol	24.5		42.0		36.3	
	3-methyl-2-cyclohexen-1-one	10.9	96.7	8.8	96.4	8.5	97.5

Cyclooctene^a^	cis- epoxide	53.0		37.0		28.0	
	2-cyclooctenone	—	53.0		37.0	10.0	38.0

Limonene^a^	cis-1,2 epoxide	34.4		21.0		18.0	
	trans-1,2 epoxide	16.8		12.3		9.4	
	limonene alcohol^f^	33.5^h^		54.0^g^		46.9^i^	
	limoneme keton*e* ^j^	9.0	93.7	12.5	99.8	8.0	82.3

Styrene^b^	epoxide	25.0		7.5		3.0	
	phenyl-acetaldehyde	1.6		7.0		1.6	
	benzaldehyde	20.0	46.6	35.0	49.5	45.0	49.6

	trans-epoxide	47.7		41.9		42.0	
Methyl-styrene^b^	methyl-benzyl-alcohole	4.5		—		8.0	
	methyl-benzyl-ketone	—		11.3		—	
	benzaldehyde	45.0	97.2	40.0	93.2	15.8	65.8

Cis-stylbene^b^	cis-epoxide	9.5		15.8		2.4	
	trans-epoxide	30.0		—		23.0	
	stylben-cetone	—		—		3.0	
	benzaldehyde	14.0	53.5	36.0	51.8	20.0	48.4

Cyclohexane^a^	cyclohexanol	5.0		7.7		4.6	
	cyclohexanone	3.0	8.0	4.4	12.1	2.4	7.0

^a^Conditions: ratio of catalyst : oxidant : substrate= 1 : 20 : 1000. ^b^Conditions: ratio of catalyst : oxidant : substrate= 1 : 50 : 1000. ^c^Reactions were completed within 12 h in *t* *e* *r* *t*-amylalcohol with H_2_O_2_ as oxidant. ^d^Reactions were completed within 4 h in CH_3_CN with H_2_O_2_ as oxidant. ^e^Reactions were completed within.1 h in CH_3_CN with *t* *e* *r* *t*-BuOOH as oxidant. ^f^Limonene alcohols were found to be a mixture of 1-ol, 2-ol, and 6-ol. ^g^54% yield corresponds to 23% for 1-ol, 13.5% for 2-ol, and 17.5% for 6-ol. ^h^33.5% yield corresponds to 9.0% for 1-ol, 6.5% for 2-ol, and 18.0% for 6-ol. ^i^46.9 yield corresponds to 26.0% for 1-ol, 8.97% for 2-ol, and 11.93% for 6-ol. ^j^The only observed ketone is the 6-one.
